# Adoption and implementation of robotic colorectal surgery using structured training approach: an experience from tertiary referral center

**DOI:** 10.1007/s13304-025-02376-x

**Published:** 2025-09-02

**Authors:** Mahmood Al Dhaheri, Reem Mubarak, Ali Toffaha, Noof Al Naimi, Ayman Abdelhafiz Ahmed, Mohamed AbuNada, Amjad Parvaiz

**Affiliations:** Colorectal Surgery, Hamad Medical Corporation, Doha, Qatar

**Keywords:** Colorectal surgery, Training, Robotic surgery, Short-term outcomes

## Abstract

Robotic colorectal surgery is rapidly growing field. It offers potential benefits over laparoscopy and these benefits are best realized with proper training. This study reports the outcomes of our first 150 consecutive cases of robotic colorectal surgery following standardized training program. Prospectively collected data for the first consecutive 150 robotic colorectal surgery procedures were analyzed for short-term outcomes. The training program followed the curriculum of the European Academy for Robotic Colorectal Surgery (EARCS) which included theoretical knowledge, online simulator training, console and bedside skills training under direct expert supervision, and objective assessment using the Global Assessment Score (GAS) form. 133/150 (89%) cases were for colorectal cancer with 61% of cases performed for rectal cancer. Two-thirds of the patients were male, median age of 54 years (range 29–87) and BMI of 28.5 (range 21–57). All procedures (*n* = 150) were performed by three surgeons. There were no mortalities or conversions to open or laparoscopy. Anastomotic leak rate was 1.3% and the overall R0 resection was achieved in 95.5% of cases. Standardized training program expedited the safe adoption of robotic colorectal surgery in our center with satisfactory oncological and clinical short-term outcomes. This study contributes valuable data on the safe adoption of robotic colorectal surgery and the application of standardized training program in the Middle East and other regions.

## Introduction

Since its introduction in the last three decades, laparoscopic colorectal surgery has gained wide acceptance and studies including randomized controlled trials have shown that laparoscopic surgery is associated with better short-term outcomes like hospital stays, fewer complications, and less postoperative pain, and good oncological outcome [1, 2]. Despite these advantages, there are known limitations to laparoscopy such as restricted movement of instruments in confined anatomical spaces, lack of camera control, and ergonomic fatigue during the performance of prolong procedures [3–5]. The introduction of three-dimensional (3D) imaging in laparoscopy has helped improving the visualization limitation; however, instability of camera control remains a challenge especially in performing complex tasks [6–9].

The introduction of robotic technologies in colorectal surgery has helped overcome some of these limitations by further improving the precision of colorectal procedures aided by sophisticated instruments, stable 3-D views from a surgeon-controlled camera, angulated instruments with seven degrees of freedom, markedly improved ergonomics, and tremor filtering [10]. These cited advantages have led to the increasing adoption of robotic surgery across many surgical specialties over the last decade. It is increasing application within the colorectal speciality, in particular rectal cancer surgery [11]. The value of robotic colorectal surgery is also evident from the increasing number of yearly publications on the subject since the first robotic colectomy was performed by Weber in 2002 [12–16]. In addition to its technical advantages, there is emerging evidence that robotic rectal surgery reduces morbidity and improves R0 rates [17]. Like any new technique, it is important to ensure that the adoption of robotic colorectal surgery is achieved safely and sensibly, to minimize potential harms and complications that are associated with learning curve. This may include careful patients’ selection, as well as training the surgeons and other medical staff on the use this new technology. A structured training program on robotic rectal surgery both in Europe and USA has facilitated the learning of this technique in an environment that promotes patient safety and enhances patient outcomes. With over 7000 robotic systems across the world, it is foreseeable that robotic approach will become part of routine surgical practice [18].

This study aims to address our experience in applying the principles of structured training program while adopting robotic colorectal surgery at a tertiary referral center in Qatar. It reports early clinical outcomes for the first 150 consecutive cases performed at our center using such approach.

## Methods

### Study population, inclusion, and exclusion criteria

Prospective data on 150 consecutive patients who underwent robotic colorectal procedures from September 2020 to August 2024 were analyzed. All patients with a biopsy proven diagnosis of rectal cancer were offered robotic surgery. Additionally, selective patients with sigmoid colon cancers and benign pathology were also offered robotic surgery. The patients had informed consent for the robotic procedure and were allowed to opt out to another modality such as laparoscopic approach.

In our practice, all patients with colorectal cancer underwent preoperative staging and were discussed in our weekly colorectal MDT meeting. Patients with T4 rectal cancer, node-positive disease, and involved or a threatened circumferential mesorectal fascia margin of < 2 mm on MRI scans had neoadjuvant therapy which involves long course chemoradiation regime with surgery performed at 12 weeks following the completion of down-staging. The presence of extramural venous invasion (EMVI) on MRI scans mandated treatment with the total neoadjuvant treatment (TNT).

Fourth generation multiport Da Vinci Xi® robot was used to perform robotic resections.

### Trainees

The three surgeons enrolled in this study had completed an average of five-year residency program followed by four years of fellowship and practice in colorectal surgery. They were exposed and were competent to perform both intermediate to advance laparoscopic colorectal procedures. The resection performed laparoscopically included right and left dissections (excluding TME for rectal cancer); on an average each one of the delegates had performed about 30 laparoscopic colorectal resections procedures independently before they embark on robotic colorectal training.

### Robotic training curriculum

Three surgeons with prior laparoscopic colorectal experience were enrolled in the training program. The training program adopted the European Academy for Robotic Colorectal Surgery (EARCS) model [19] and led by expert robotic surgeon (AP) with experience of more than 1500 robotic cases and worldwide experience in teaching and training colorectal surgery both robotic and laparoscopic surgery.

Each trainee went through a series of assessment-based modules to gain foresight into the training pathway. This was subsequently augmented with a practical hands-on clinical component. The curriculum included the following modules:

1. Theoretical knowledge development using lectures and cadaveric videos covering the pelvic anatomy and anatomy related to Total Mesorectal Excision (TME) [20, 21].
2. Online Training on the robotic system as per Intuitive curriculum (https://www.intuitive.com/en-us/products-and-services/da-vinci/learning)
3. Training on Da Vinci simulator in our institute (two simulators available in our training center ITQAN simulation center https://www.hamad.qa/EN/Education-and-research/SIM/Pages/default.aspx)
4. Case observation for colonic and rectal resections done by the senior robotic proctor, each trainee – on average – had 5 case observation.

Each one of the trainees were expected to fulfill the above pre-requisites before embarking on hand on training under direct supervision. On average 30 h of simulated training (in designed center in the country belong to Hamad Medical Corporation called ITQAN https://www.intuitive.com/en-us/products-and-services/da-vinci/learning) was achieved by each of the trainees completing competence score in each module of simulation, in parallel to bedside assistance.

Once the trainees complete the above pre-requisites, they proceed to hands-on training under direct supervision of the trainer (AP). The training commences as per modules described in EARCS and assessed using the Global Assessments Score form (GAS) [19].

**Fig. 1:**
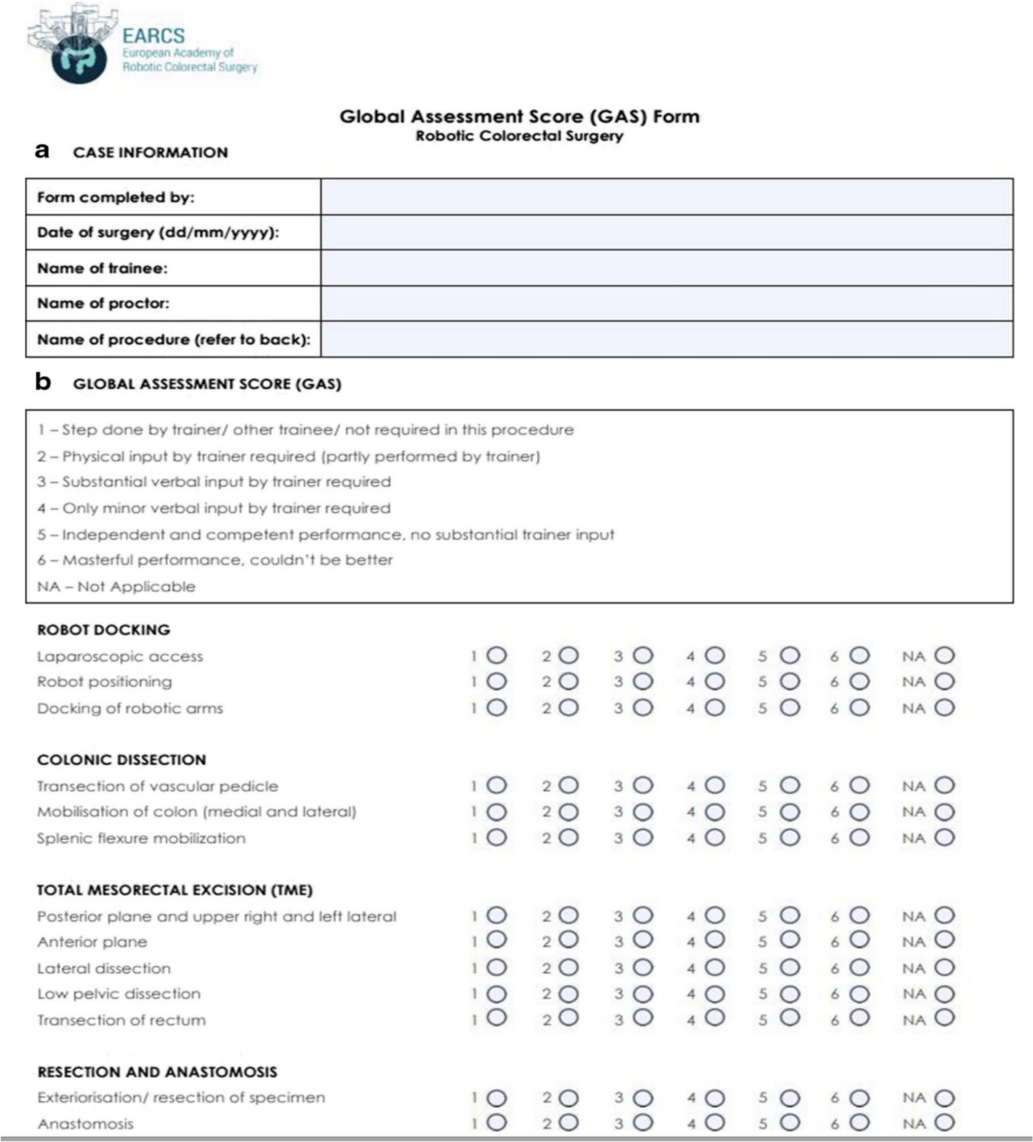
GAS form

The EARCS contains four modules, each containing several components. These include (1) robotic docking, (2) colonic dissection, (3) TME, and (4) resection and anastomosis. In Fig. 1 we present the GAS form used by the EARCS faculty, including the components of each module. Using the GAS form, each component is scored from 1 to 6 (or not applicable if the step is not performed) with the scores given corresponding to the competence levels presented in the GAS form in (Fig. 1). As demonstrated, the higher the score, the higher the competence level for each component. Each supervised operation was scored in real time by the proctor.

On an average the trainees performed a median of 18 supervised procedures the duration of that range between 30 and 40 weeks on average before they were assessed for competence and were allowed to perform independent procedures. However, the challenge remained in difficult low rectal cancer where they occasionally needed help from senior robotic surgeon.

### Statistical analysis

Using Microsoft Excel 2016™ for the statistical analysis, the data are expressed as median with range for non-parametric data and mean with standard deviation for parametric data.

## Results

Between September 2020 and August 2024, a total of 150 patients underwent robotic colorectal surgery using da Vinci Xi system. All patients were operated in a single center by three surgeons.

### Baseline characteristics

There were 102 (68%) male patients, median age was 54 years (range 29–87), and median body mass index (kg/m2) was 28.5 (range 21–57). 133 (89%) procedures were performed to treat cancer, while the remaining 17 (11%) were for benign conditions. 61% of the cancer cases were rectum and 34.5% were left colonic cancers. 74% of the rectal cancer received neoadjuvant treatment. For the 17 benign cases, six of them were complicated diverticulitis who underwent sigmoidectomy, four cases were ulcerative colitis, two of whom underwent restorative proctocolectomy and the rest had completion proctectomy with end ileostomy. Four patients had excision of retrorectal cyst (Table 1).

**Table 1:** Patients characteristics and indications for surgery

Characteristic	*n* (150)	%
Males	102	68%
Females	48	32%
Age median (years) (range)	54 (29–87)	
BMI median (kg/m^2^) (range)	28.5 (21–57)	
ASA		
I	11	7.3%
II	94	62.6%
III	44	29.3%
IV	1	0.6%
Indications for surgery		
Cancer	133	88.6%
Rectal cancer	81	61%
Left side colon cancer	46	34.5%
Right colon cancer	6	4.5%
Benign pathology	17	11.4%
Diverticulitis	6	35.2%
UC*	4	23.5%
FAP	1	5.8%
Excision of retro rectal cyst	4	23.5%
Reversal of Hartman	1	5.8%
Repair of Pouch-vesical fistula	1	5.8%
Neoadjuvant radiotherapy (Cancer of rectum)	60 (out of 81 rectum)	74%
Procedure		
Low anterior resection (TME)* with loop ileostomy	54	36%
Abdominoperineal excision (APE)	26	17.3%
Sigmoidectomy, left hemicolectomy and AR*	46	34.6%
Right hemicolectomy	6	4%
IPAA*	3	2%
Completion proctectomy and end ileostomy (benign)	3	2%
Excision of retro rectal cyst	4	2.6%
Reversal of Hartman	1	0.6%
Repair of Pouch-vesical fis	1	0.6%

^*^UC Ulcerative colitis, *TME* Total mesorectal excision, *AR* Anterior resection, *IPAA* Ileal pouch anal anastomosis, *FAP* Familial adenomatous polyposis

### Perioperative and postoperative outcomes

Procedures performed included 54 (36%) low anterior resections with diversion loop ileostomy, 26 (17%) abdominoperineal excision (APE), 52 (35%) sigmoid colectomy, 6 (4%) right hemicolectomy, 3 IPAA, and 3 completion proctectomy with end ileostomy (Table 1). The median length of hospital stay was 6 days (range 1–53). There was no mortality and no conversion to either open or laparoscopic surgery. Two patients required reoperation: one case of anastomotic leakage post-sigmoidectomy for sigmoid cancer required laparoscopic exploration, abdominal lavage, and creation of diversion loop ileostomy, and the other patient had low anterior resection with loop ileostomy for rectal cancer developed anastomotic leakage and underwent laparoscopic lavage. There were two cases of readmission within 30 days of surgery for stoma-related obstruction (Table 2).

**Table 2:** Short term clinical and oncological outcomes

	*n* (150)	%
Length of stay days median (range)	6 (1–53)	
Conversion to open or laparoscopic	0	0
30-day mortality	0	0
30-day readmission	2	1.3%
30-day reoperation	2	1.3%
30-day morbidities	14	9.3%
Superficial SSI	6	4%
Anastomotic leakage	2	1.3%
Ureteric injury	2	1.3%
Ileostomy related obstruction	6	4%
Intra-abdominal hematoma	1	
Lymph node yield median (range)	14 (0–36)	
Margins		
R0 (total cancer)	127/133	95.5%
R1 (total cancer)	6/133	4.5%
R0 (rectum)	75/81	93%
R1 (rectum)	6/81	7%
R2	0	
pTNM Stage		
0 (pCR) rectum	13 (out of 81 rectum)	16%
Total staging for all cancers		
I	27	20.3%
II	34	25.5%
III	43	32.3%
IV	16	12%

^*^SSS Surgical site infection, *pCR* Pathological complete response

Two patients (1.3%) suffered ureteric injury. Both cases were APE and the injury happened during the perineal part of the procedure.

### Oncological outcomes

For the rectal cancer, out of 81 rectal cancer patients, 54 (67%) underwent low anterior resection with diversion loop ileostomy and 26 (32%) had abdominoperineal excision (APE). 74% received neoadjuvant treatment in the form of long course chemoradiation or total neoadjuvant treatment. R0 resection achieved in 93%, while 7% had R1 resection most of them in the form of microscopic involvement of radial margin. 16% reported complete pathological response.

For the colon cancer cases, out of 52 cases, 46 cases were performed for sigmoid and descending colon cancer and the other 6 cases were for right colon cancer. R0 resection was achieved in all the cases.

The overall median lymph node yield for all cancer group was 14 (0–36) (Table 2).

## Discussion

The safe adoption of robotic colorectal surgery requires structured training for the surgeons and the support staff [19, 22–25]. This includes not only learning the surgical techniques but also the preoperative planning, patient selection, and postoperative care. In this study we have demonstrated that through the application of a structured training pathway, robotic colorectal surgery can be safely adopted in Qatar with good short-term outcomes.

The need for standardized structured robotic training was identified in the in US and Europe which resulted in several training programs, mostly emerging from Urology [22]. In the US, the Fundamentals of Robotic Surgery curriculum (FRS) has proven to be an effective program and those enrolled in the program showed better performance than controls (the program has been created as an open-source course and its freely available at www.frsurgery.org) [26]. Similarly, the European Academy of Robotic Colorectal Surgery (EARCS) run a successful training program based on objective assessment tools and included 26 training centers across Europe. The short-term clinical outcomes were consistently good, and the trainees safely achieved short-term surgical outcomes comparable to their trainers and overcome the learning process in a way that minimizes patient harm while achieving competency. Other groups have successfully either adopted these programs or designed their own curriculum [27, 28].

In our study, we implemented the EARCS program where the core curriculum is similar to the laparoscopic program for which we had prior experience.

The clinical short-term outcomes observed in our study are consistent with findings in the wider literature. The rate of anastomotic leakage was 1.3%, and there were no conversions to open or laparoscopic procedures, similar to the EARCS study which showed figures of 3% and 2%, respectively [19]. Regarding the oncological short-term outcomes, overall R0 resection was achieved in 95.5% of cases, and a median of 14 harvested lymph nodes (range: 0–36) in line with the literature reports [19, 27]. Six patients with locally advanced tumors requiring neoadjuvant treatment followed by resection had R1 resection. Four of them were APE and the other two cases were low anterior resection.

Additionally, our data show 1.3% readmissions and no mortality within 90 days. The median length of hospital stay was 6 days, and the rate of surgical site infection was 4%, consistent with reported literature [23, 29].

Two patients suffered a ureteric injury during APE. Both were young patients < 45 years old with a locally advanced low rectal cancer ypT4a ypN2b M0, who underwent APE following a poor response to total neoadjuvant chemoradiotherapy with the injury occurred during the perineal part of the procedure.

Notably, most of our patients were relatively young, with a median age of 53 years (range: 29–87) and a median BMI of 28.5 (range: 21–57). Despite the challenges associated with higher BMI and male gender, both known to complicate surgery, we observed favorable outcomes in these patients.

The strength of our study comes from the nature of a prospectively maintained database and includes all the consecutive patients. Additionally, we believe that it is the first reported study from the Middle east that highlights the importance of both standardized as well as structured training before embarking on independent clinical practice. The standardization of techniques and training pathway results in reproducible good outcomes. This can lead the way for other centers in the region to successfully adopt robotic colorectal surgery.

Our study suffers from limitations which include a small sample size and lack of the functional data – bowel function, urological, and sexual function – as well as long-term oncological outcome. Another limitation may be the mixture of cases, benign and cancerous, rectums, and colons. However, the results of the study were consistent to those of larger studies and the subgroup analysis was impractical since the sample size was small.

Nevertheless, these constraints do not compromise the core message of our study which focus on standardizing the training that leads to safe implementation of robotic program in colorectal surgery unit with short-term outcomes that are in line with published literature.

## Conclusion

Our study demonstrates that standardization of robotic colorectal surgery training leads to safe and successful adoption with good short-term outcomes. This study can serve as a model to safely implement robotic colorectal surgery in the region and globally. However, additional studies, specifically those including functional and oncological long-term data, are necessary to prove long-term advantages of this approach.

## Data Availability

The data are available upon request.
